# Predictable Words Are More Likely to Be Omitted in Fragments–Evidence From Production Data

**DOI:** 10.3389/fpsyg.2021.662125

**Published:** 2021-07-22

**Authors:** Robin Lemke, Ingo Reich, Lisa Schäfer, Heiner Drenhaus

**Affiliations:** ^1^Collaborative Research Center 1102, Saarland University, Saarbrücken, Germany; ^2^Department of Modern German Linguistics, Saarland University, Saarbrücken, Germany; ^3^Department of Language Science and Technology, Saarland University, Saarbrücken, Germany

**Keywords:** information theory, fragments, ellipsis, script knowledge, surprisal

## Abstract

Instead of a full sentence like *Bring me to the university* (uttered by the passenger to a taxi driver) speakers often use fragments like *To the university* to get their message across. So far there is no comprehensive and empirically supported account of why and under which circumstances speakers sometimes prefer a fragment over the corresponding full sentence. We propose an information-theoretic account to model this choice: A speaker chooses the encoding that distributes information most uniformly across the utterance in order to make the most efficient use of the hearer's processing resources (Uniform Information Density, Levy and Jaeger, 2007). Since processing effort is related to the predictability of words (Hale, 2001) our account predicts two effects of word probability on omissions: First, omitting predictable words (which are more easily processed), avoids underutilizing processing resources. Second, inserting words before very unpredictable words distributes otherwise excessively high processing effort more uniformly. We test these predictions with a production study that supports both of these predictions. Our study makes two main contributions: First we develop an empirically motivated and supported account of fragment usage. Second, we extend previous evidence for information-theoretic processing constraints on language in two ways: We find predictability effects on omissions driven by extralinguistic context, whereas previous research mostly focused on effects of local linguistic context. Furthermore, we show that omissions of content words are also subject to information-theoretic well-formedness considerations. Previously, this has been shown mostly for the omission of function words.

## 1. Introduction

In order to communicate a message to a hearer, speakers have to select a particular utterance from a set of utterances that can be used to convey this message in the utterance situation. Besides utterances that contain different word forms or syntactic constructions, speakers can often resort to a subsentential utterance like (1-a) instead of a full sentence like (1-b). Despite their reduced form, given an appropriate context, such subsentential utterances are interpreted as roughly meaning-equivalent to their fully sentential counterparts.


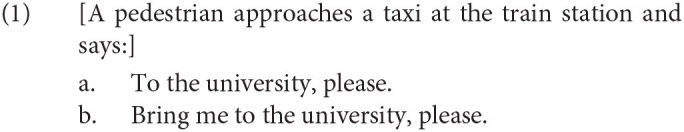


Subsentential utterances, or fragments^1^ (Morgan, 1973), have been discussed extensively in the theoretical literature from a syntactic perspective, in particular with respect to the question of whether they are a genuinely nonsentential output of syntax (Ginzburg and Sag, 2000; Barton and Progovac, 2005; Culicover and Jackendoff, 2005; Stainton, 2006), or derived by ellipsis from regular sentences (Merchant, 2004; Reich, 2007; Weir, 2014).

Only a few studies have looked into the questions of why speakers use fragments at all, and under which circumstances they prefer them over the corresponding full sentence. In the theoretical literature, the grammaticality of omissions has been related to information structure, in particular to the notions of focus and givenness (Merchant, 2004; Reich, 2007; Weir, 2014; Ott and Struckmeier, 2016; Griffiths, 2019). Leaving aside conceptual differences between these accounts, overall they agree on the prediction that only material that is given in an information-structural sense (Schwarzschild, 1999) can be omitted and that words that belong to the focus (see e.g., Rooth, 1992) must be realized. Such information structure-based accounts however explain only why fragments can or cannot be used under particular conditions, but not why they are (not) used when they are licensed by grammar.

The sparse evidence available with respect to the actual usage of fragments suggests that the choice between a fragment and a sentence is driven by the general tendency to maximize communicative efficiency: Speakers adapt the form of the utterance to properties of the situation and the hearer. This idea has been formalized in information-theoretic (Levy and Jaeger, 2007; Levy, 2008) and game-theoretic frameworks (Franke, 2009; Frank and Goodman, 2012). Bergen and Goodman (2015) combine a game-theoretic model of rational communication with a noisy channel model, which in sum predicts that the choice between a fragment and a complete sentence is based on a trade-off between the cost for producing an utterance and the risk of not being understood correctly. Even though the account is promising, it is only illustrated with a highly simplified example of a question-answer pair. Bergen and Goodman (2015) do not apply it to more realistic communication situations which involve more diverse utterances, potentially communicated messages and predictability effects drive by extralinguistic context. Lemke et al. (2021) in turn explain the choice as adaptation to the processing resources of the hearer. They argue that predictable utterances, which require less processing effort (Hale, 2001; Demberg and Keller, 2008; Levy, 2008), are more often reduced in order to use the hearer's processing resources efficiently.

Both Bergen and Goodman (2015) and Lemke et al. (2021) provide an explanation for when speakers prefer to reduce their utterance more strongly and consequently to produce a fragment rather than a full sentence, but they do not make clear predictions about why speakers prefer *particular* fragments if a sentence can be reduced in different ways. For instance, in the taxi example (1), it seems more natural for the passenger to say *to the university* than *me the university*, even though both of these fragments reduce the utterance to a similar extent. While Lemke et al. (2021) just show that the reduction of predictable utterances is more acceptable, Bergen and Goodman (2015) include a cost term in their model that penalizes utterances that are effortful to produce. Since Bergen and Goodman (2015) derive a preference for fragments from this cost term, it is most likely intended to be affected by the length of an utterance, but they do not make this explicit or discuss other sources of production effort, like a cost for retrieving unpredictable words (Ferreira and Dell, 2000). In the absence of more specific accounts of why particular words are more likely to be omitted, the general tendency to densify predictable utterances or those produced in the absence of noise cannot fully explain speakers' production choices.

In this article we propose an information-theoretic account of fragment usage, according to which the predictability of utterances and words therein determines (i) whether speakers choose a sentence or a fragment to get their message across, and, in the latter case, (ii) which words are omitted in fragments. We hypothesize that this choice is driven by the tendency to distribute processing effort uniformly across the utterance (Fenk and Fenk, 1980; Levy and Jaeger, 2007). Since the effort required to process a word is inversely proportional to its probability (Hale, 2001; Levy, 2008), our account makes two specific predictions: First, likely words are preferably omitted, and second, words that increase the likelihood of otherwise unlikely following words are preferably inserted.

An information-theoretic approach is particularly promising for two reasons: First, Lemke et al. (2021) found that fragments are overall more strongly preferred in predictive contexts. This finding is in line with our account, because in predictive contexts the words within an utterance are overall more likely and thus also more likely to be omitted. However, Lemke et al. (2021) did not look into the more fine-grained predictions of the information-theoretic account on the word level. Second, information-theoretic constraints have been shown to explain the distribution of omissions particularly on the word level, such as those of complementizers, pronouns, articles and case markers (Levy and Jaeger, 2007; Frank and Jaeger, 2008; Jaeger, 2010; Asr and Demberg, 2015; Kurumada and Jaeger, 2015; Norcliffe and Jaeger, 2016; Lemke et al., 2017). Even though most of these studies focused on semantically relatively empty function words,^2^ information-theoretic constraints make similar predictions on the omission of content words. Therefore, our research extends previous evidence for information-theoretic considerations on the speaker's preferences on optional omissions to content words and larger phrases.^3^

Testing the predictions of information-theoretic constraints on optional omissions in general requires (i) a corpus that contains instances of the relevant omissions and the corresponding full forms (in our case, complete sentences), (ii) knowing *which* expressions have been omitted, and (iii) a method to estimate the probability of both the omitted and the realized expressions in the data. Logistic regressions can then show whether the predictability of a target expression given the material surrounding it has a significant effect on the likelihood of the omission of the target expression. The application of this procedure to fragments however is difficult due to three properties of fragments that inhibit probability estimation with standard language models. We address these challenges by collecting a data set with a production task that allows us to investigate the predictions of our account.

1. It is often unclear which lexical item has been omitted in fragments. For instance, in the taxi example, a hearer who processes the fragment *to the university* in (1-a) can interpret it as *bring me to the university*, or *I'd like to go to the university*, and which of these reconstructions is assumed obviously affects the words' probability estimates. In order to reduce this ambiguity, we preprocess our data set so that that omissions can be relatively unambiguously resolved.
2. Fragments often occur discourse initially, so that the likelihood of utterances and words therein is determined by extralinguistic context, which language cannot take into account, because this information is not contained in standard text corpora. We quantify effects of extralinguistic context by eliciting the utterances in our data set with script-based context stories, which are based on probabilistic event chains extracted from DeScript (Wanzare et al., 2016), a freely available crowd-sourced corpus of script knowledge.
3. Levy and Jaeger (2007) observe a circularity issue that concerns probability estimations on elliptical data: If predictable expressions are particularly often omitted, they will appear to be rare in a corpus, or at least not as frequent as their probability would suggest, just *because* of their high ratio of omission. We propose a new approach to estimate the probability of each word in our data that is not vulnerable to the circularity issue. Our method relies on a version of the data set that does not contain omissions, so probability estimation is not affected by a word's actual omission rate.

Our study is the first empirical investigation of why speakers choose (not) to omit particular words in fragments, and consequently, in which situations they prefer to use a fragment rather than a full sentence. From the broader perspective of information-theoretic research on language, we extend previous evidence for information-theoretic processing constraints in two ways. First, we provide evidence for such effects on content words, and second, we find that not only local linguistic context, but also extralinguistic context drives predictability effects. From a methodological perspective, our probability estimation method circumvents the circularity issue observed by Levy and Jaeger (2007) and provides an approach to quantifying by-word probability in the presence of omissions.

The article is structured as follows: Section 2 sketches our information-theoretic account and its central predictions on fragment usage. Section 3 presents the production experiment and section 4 summarizes our main results and contributions and their relevance for related theories of probability effects on optional omission and reduction.

## 2. An Information-Theoretic Account of Fragment Usage

Information-theoretic processing constraints have been shown to explain the distribution of a wide range of reduction phenomena. Their application ranges from phonological reduction (Bell et al., 2003, 2009; Aylett and Turk, 2004; Tily et al., 2009; Demberg et al., 2012; Kuperman and Bresnan, 2012; Seyfarth, 2014; Pate and Goldwater, 2015; Brandt et al., 2017, 2018; Malisz et al., 2018) to morphological effects on contraction (Frank and Jaeger, 2008) and case marker omission (Kurumada and Jaeger, 2015; Norcliffe and Jaeger, 2016) to pronominalization (Tily and Piantadosi, 2009), and, what is most closely related to omissions in fragments, optional omissions of various types of function words (Levy and Jaeger, 2007; Jaeger, 2010; Asr and Demberg, 2015; Lemke et al., 2017) and preverbal subjects (Kravtchenko, 2014; Schäfer, 2021).

The central idea of information-theoretic accounts of omission phenomena is that speakers use omissions in order to optimize their utterance with respect to properties of the situation and the hearer. Information-theoretic approaches model this as the *channel capacity* in the sense of Shannon (1948), i.e., the maximum amount of information that can be transmitted across a channel with a limited capacity. *Information*, or *surprisal* (Hale, 2001) is defined probabilistically as −log_2_ *p*(*word* | *context*), i.e., the negative logarithm of a word's likelihood to appear in a given context. The less likely a word is, the more information it conveys. Since Hale (2001), this notion of information has been related to processing effort: The more information a word conveys, the more processing effort it requires (see also Hale, 2006; Demberg and Keller, 2008; Levy, 2008). Given the link between information and processing effort, we interpret channel capacity as an upper bound to the cognitive resources of the hearer. If this upper bound is exceeded, the hearer is not able to successfully process an input, whereas under-utilizing channel capacity results in inefficient communication.^4^ Taken together, this predicts that speakers adapt their utterance so as to communicate at a rate close to, but not exceeding, channel capacity. Information maxima that exceed channel capacity shall be avoided, just like information minima that do not make use of the full cognitive resources available to the hearer.

**Figure 1:**
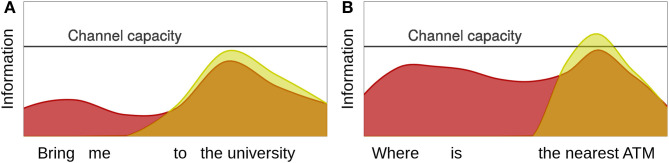
Hypothetical ID profiles for the predictable **(A)** and the unpredictable **(B)** sentence (red) and fragment (yellow) examples in the taxi example. In the case of the predictable utterance the fragment is more well-formed, because it fragment avoids the trough in the profile caused by *bring me*. In the case of the unpredictable utterance the sentence is more well-formed, because the fragment causes a peak in the profile that exceeds channel capacity.

UID makes two main predictions with respect to omissions in fragments. Words that are more likely in context are more likely to be omitted in order to **avoid local information minima** which result in the underutilization of the hearer's processing resources and appear as *troughs* in the information density (ID) profile, as the left facet of Figure 1 shows. In the taxi example in (1), it is very likely that the pedestrian approaching the vehicle wants to be brought somewhere, hence the words *bring me* are highly predictable and convey only little information. In contrast, the destination is less predictable in this context, hence the information on *the university* is higher. Such information minima are inefficient and can be smoothed by omitting these words. In situations where the structure resulting from this omission is a fragment, UID hence predicts that speakers prefer this fragment over the corresponding full sentence.

In contrast, words that precede unpredictable words are more likely to be realized in order to **avoid local information maxima**, which exceed the hearer's processing resources and appear as *peaks* in the ID profile. Inserting optional words often increases the predictability of following ones, because this restricts the range of possible successors (Hale, 2001; Levy, 2008). Consequently, inserting these words can reduce the information maximum on following words.^5^ For instance, in the taxi example the pedestrian could ask for the nearest ATM instead of asking for a ride. If asking for a direction is less likely, the words *where is* will be more informative and hence less likely to underutilize the hearer's processing resources. Furthermore, if *the nearest ATM* is less likely to be a potential destination than the university, it will be more likely to yield a peak in the ID profile that exceeds channel capacity. Inserting *where is* in turn might increase the likelihood of locations that are asked for frequently, like a subway station, a bus stop or an ATM, and thus smooth the peak on *the nearest ATM* that occurs in the fragment. Hence, in case of this example, a speaker should prefer to produce a complete sentence rather than a fragment.

An important property of UID is that the omission or insertion of optimal is limited to variation between “the bounds defined by grammar” (Jaeger, 2010, p. 25): Omissions which are ruled out by grammar will not be preferred even if they distribute information more uniformly across the signal. For instance, Schäfer (2021) finds UID effects on the omission of preverbal subjects in German text messages, however, in more prototypically written text types in her corpus there is not a single instance of this construction. With respect to fragments, this predicts that omissions are restricted to those that are available in the language and text type under investigation.

Determining whether a specific omission contributes to the optimization of an utterance given these predictions in principle would require knowing channel capacity. Only information maxima which exceed channel capacity are to be avoided. In practice however, channel capacity is necessarily unknown, since the amount of processing resources that are available to the hearer depends on properties of the situation (Engonopoulos et al., 2013; Häuser et al., 2019) and of the individual hearer (Pate and Goldwater, 2015). Interlocutors must therefore infer channel capacity. This assumption is also psychologically plausible, because we can never precisely know the amount of processing resources that is available to a hearer. In consequence, our hypothesis pertains even if channel capacity is unknown: Predictable words are more likely to yield an information minimum, which is smoothed by omission, and unpredictable ones are more likely to yield an information maximum, which can be reduced by the insertion of preceding material.

## 3. Production study

We use a production task to collect a data set that is suitable for the investigation of the predictions of the information-theoretic account: In order to avoid troughs and peaks in the ID profile, speakers prefer to omit predictable words and to insert additional redundancy before unpredictable words. Such a data set must (i) contain both instances of such omissions and of the corresponding full forms, (ii) allow for the quantification of predictability effects driven by extralinguistic context, and (iii) it must allow for the unambiguous reconstruction of the omitted material, because the way in which omissions are reconstructed affects the estimation of individual words' surprisal. In order to control for extralinguistic context, we elicited our data set with 24 script-based stories, as we describe at detail in sections 3.1, 3.2. In section 3.3 we discuss how we pre-processed the data in order to ensure a relatively unambiguous reconstruction of omitted material. Section 3.4 describes our surprisal estimation methods and section 3.5 the statistical analysis of the data.

### 3.1. Materials

In order to control and quantify effects of extralinguistic context, we used 24 stories like (2) to elicit participants' responses. We conducted the study in German and translate materials presented here for convenience. Participants were asked to produce the most likely utterance to be produced by the specified person in the situation described in the story. For each story, we collected a total of 100 responses. Since all of these responses are produced in the same context, this approach allows us to quantify effects of extralinguistic context on the likelihood of a response and the words therein.


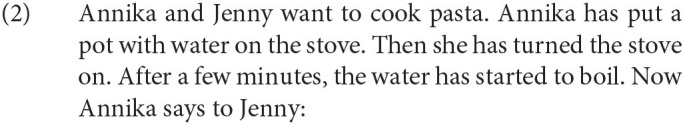


Stories like (2) might in principle trigger different expectations in different subjects, depending on their experiences and world knowledge. In order to minimize such effects, we based our stories on scripts, i.e., knowledge about the stereotypical time-course of everyday activities that is represented by partially ordered sequences of events (Schank and Abelson, 1977). For instance, the script about cooking pasta that underlies (2) contains events like *put a pot on the stove, turn the stove on* and *wait for the water to boil*, which most of the time appear in this order. Psycholinguistic studies have shown that script events prime upcoming events within the same script (see e.g., Bower et al., 1979; McKoon and Ratcliff, 1986; Millis et al., 1990; van den Broek, 1994; van der Meer et al., 2002; Nuthmann and Van Der Meer, 2005; Bicknell et al., 2010; Delogu et al., 2018), hence we expect that our context stories trigger expectations about what happens next and consequently determine which utterance is produced. For instance, in our example in (2), a request to pour the pasta into the pot or to give the speaker the pasta seems intuitively likely, whereas a question about ingredients of the sauce might be less likely.

We based our materials on event chains extracted from DeScript (Wanzare et al., 2016), a crowd-sourced corpus of script knowledge, in order to rely on empirically founded script representations rather than on our own intuitions. DeScript is a publicly available resource that contains about 100 descriptions of the stereotypical time-course of 40 everyday activities which differ in their complexity, the degree of variation and conventionalization (e.g., *flying on an airplane, making scrambled eggs* or *taking a bath*). We used a semi-automatic approach for extracting event chains from the corpus, i.e., sequences of events that are likely to follow each other.^6^ Following Manshadi et al. (2008), we defined an event as the finite verb and its nominal complement, e.g., put pot in (2). After dependency-parsing the corpus with the Stanford parser (Klein and Manning, 2003) included in the Python Natural Language Toolkit (Loper and Bird, 2002), we extracted these event representations from it. We estimated the likelihood of an event given the previous one with bigram language models trained on the manually preprocessed data for each script with the SRILM toolkit (Stolcke, 2002). We then extracted sequences of three events that were most likely to follow each other and used these event chains to construct our materials. The first sentence in each item introduces the script (cooking pasta), and the next three ones elaborate the event chain (put pot, turn on stove, boil water). In context on this event chain, we expect a relatively high ratio of utterances referring to the most likely event to follow in the event chain, i.e., that of pouring the pasta into the pot.

### 3.2. Data Collection

The study was conducted using the LimeSurvey presentation software (LimeSurvey GmbH, 2012). The 24 stimuli were distributed across two lists (12 per list), mixed with 8 unrelated fillers that resembled our context stories and presented in individually fully randomized order. We recruited 200 self-reported native speakers of German on the crowdsourcing platform Clickworker, half of which were assigned to each of the lists. Each participant received €2 for participation. All participants agreed to the collection and aggregated or anonymized publication of their responses by participating in the study. We did not collect any personal data like participants' names, IP addresses or IDs on the Clickworker platform, whose collection would require additional data safety measures.

Subjects were asked to provide the most natural utterance to be produced by the specified person in the situation described by the context story. In the instructions we asked subjects to produce *complete sentences*. Initially, we planned to collect two data sets, one without omissions, that would be used for surprisal estimation, and one with ellipses. Since subjects however produced omissions (up to 60% of all grammatically required words in a script were omitted, see also **Figure 3**) despite having been told not to do so, we used this data set for both surprisal estimation and for analysis. This might raise the concern that the ratio of omissions might be lower than if the task would be totally unconstrained, i.e., if we asked for *any* utterance that participants perceived as likely. In order to address this, we collected a second data set consisting of 50 responses for each item following the same procedure, but asking subjects to provide the most likely *utterance* in this context. The overall rate of omissions was slightly higher in the data set collected by asking for “utterances” (25.74%) than in the data set collected by asking for “sentences” (23.79%). However, a linear mixed effects regression that compared the omissions rates for each of the items between both data sets shows that omissions are not significantly more frequent when asking for “utterances” rather than for “sentences” (χ^2^ = 0.8, *p* > .3). Therefore, we used the initially collected data set, which was twice as large, for analysis.

### 3.3. Production Data Preprocessing

**Figure 2:**
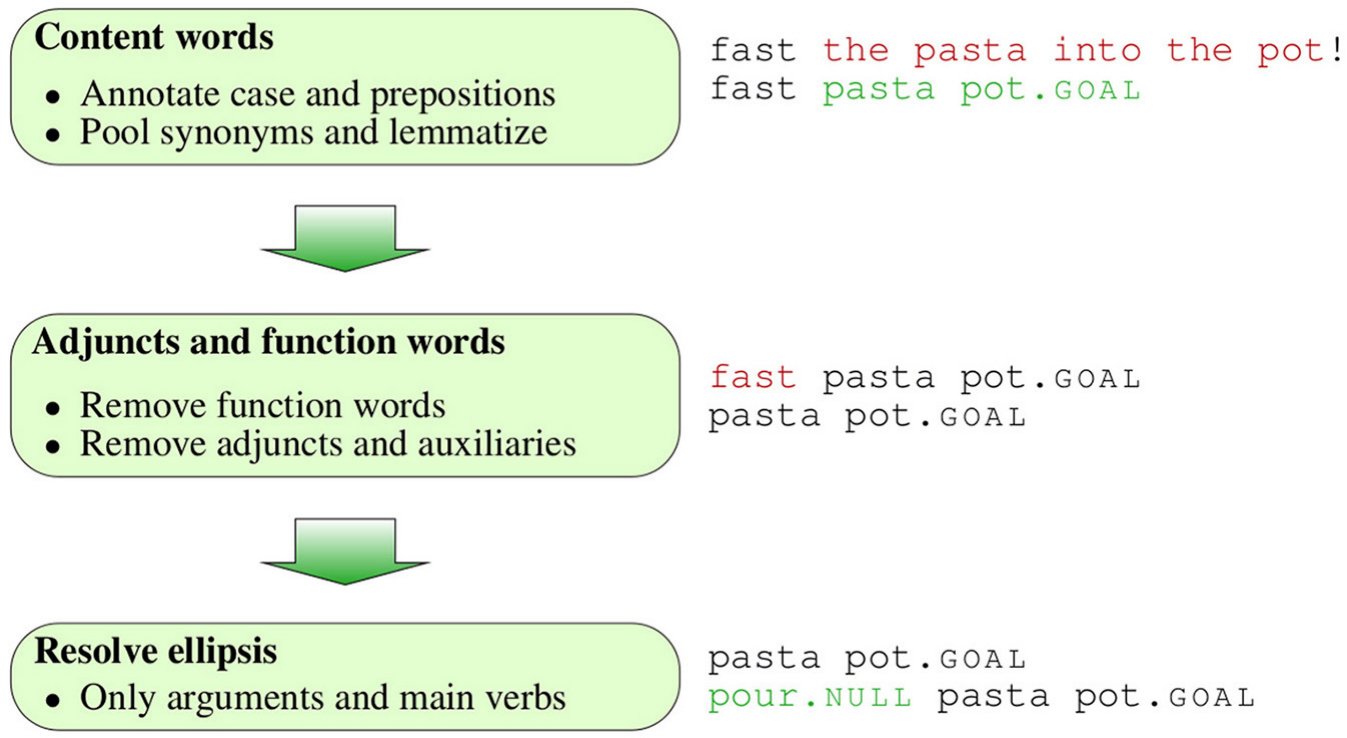
Overview of the preprocessing procedure at the example of the fragment *Schnell die Nudeln in den Topf* “The pasta into the pot, quickly!” in (3), which we illustrate at the English translation of the example. First, the NP *the pasta* and the PP *into the pot* are merged to single expressions and the information conveyed by the function words annotated on the noun. Then the adverb *fast* is removed and finally the missing verb is reconstructed.

Our preprocessing procedure had two goals. First, we standardized lexical items in order to facilitate unambiguous and homogeneous ellipsis resolution and to facilitate surprisal estimation, and second, we adapted our data to requirements of the statistical analysis with logistic regressions. Figure 2 provides an overview of the procedure for the fragment utterance in (3). The main steps, which we describe in detail in what follows, consisted in annotating information conveyed by function words and removing these, lemmatizing the remaining words, pooling synonyms to a single lemma, removing optional words, and finally manually resolving pronouns and ellipses.


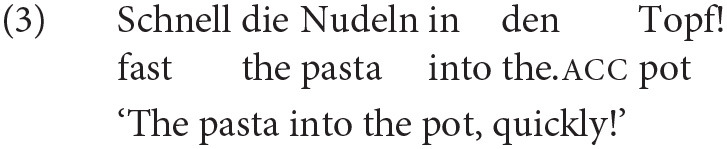


We first annotated the information conveyed by prepositions and articles as tags like acc for accusative case on the corresponding nouns and subsequently removed the function words from the data set. This step accounts for the assumption that UID explains only *grammatical* variation. Since the omission of articles is ungrammatical in standard German (Reich, 2017) and that of prepositions from PPs highly degraded (Merchant et al., 2013; Lemke, 2017), their omission appears to be blocked in German for reasons which are independent from UID. Otherwise, our logistic regression analysis, which predicts the omission of a word from information-theoretic measures, might predict that the omission of a particular article or preposition is preferred even though it is ungrammatical. For prepositions we annotated the preposition as a tag on the noun, whereas for articles we annotated only distinctive case marking encoded on the article.^7^ Annotating prepositions and case on the noun ensures that the complete phrase is treated as a single unit in the regression analysis and that the information conveyed by the removed word is preserved. Since it is an important cue toward the omitted material, for instance, encountering an accusative DP reduces the possibility of encountering another one within the same sentence (Levy, 2008).

The next step consisted in pooling synonym content words, i.e., nouns and verbs, to a single lemma. By synonyms, we understand words that refer to the same object in a single scenario: For instance, verbs like *schütten* ‘to pour' and *reintun* ‘to put inside' are no synonyms in general, but we pooled them in the pasta scenario when they were used to refer to the action of pouring something into the pot. The same holds for *the water* and *the pot* in utterances asking the hearer to put something inside the pot with the boiling water: In this context it is impossible to put something into the pot without putting it into the water and vice versa. In contrast, we did not merge categorially different order items like *hamburger, nachos* or *fries* in ordering scenarios, because they are different items at the moment in which the utterance occurs and their usage results in different outcomes of the situation. We used the most frequently occurring lexicalization in the data set as the label for an object or action in the script. Since we estimated surprisal for the data of each scenario separately, possible duplicate labels in the data for different scripts have no effect on the surprisal estimates.

Merging synonyms to a single label is necessary for two reasons. First, it facilitates the resolution of omissions: If an omitted verb in a fragment like (2) can be resolved either as *schütten* ‘to pour' or *reintun* ‘to put inside', the decision between either of these verbs would be arbitrary, but if there is only one option after pooling, resolution becomes unambiguous. Second, we use the pre-processed structures for surprisal estimation, and the presence of various synonym lemmas in the data would split the total probability mass of e.g., an action to occur among these alternatives.^8^ A further advantage of pooling is that it reduces the lexicon size in the data for each scenario and thus allows to estimate word probabilities more accurately.

We then removed all optional words from our data set, specifically adjectives, adverbials and adverbs, but also modal verbs and particles. This ensures that our data set contains only those expressions, whose absence indicates that they have been omitted. Since the data set must contain both omissions and the corresponding complete counterparts, including e.g., locative adjuncts in our analysis would imply that locative adjuncts have been omitted in all sentences that do not contain such. However, leaving predictable adjuncts implicit is not an omission that results in fragments, and hence not the type of omission that we are concerned with in this article.^9^

Finally, we resolved the reference of pronouns and reconstructed ellipses in our data. Resolving ellipsis is a prerequisite for modeling whether the words that UID predicts to be omitted are really more often omitted in the production data. We added those expressions that are minimally required in a full sentence, i.e., missing verbs and their arguments. Since we inserted the corresponding labels after pooling synonyms, ellipsis resolution was straightforward. For instance, in the case of a fragment like *The pasta into the pot!*, after pooling there is only a single verb *pour* that can be inserted to enrich the fragment to a full sentence. In what follows we refer to the annotated data set resulting from this procedure, that contains both words that were actually produced and those words that were omitted and reconstructed as the *enriched* data set. Based on this corpus, our regression analyses test for each word within this data set whether our information-theoretic predictors significantly determine its omission in the original data.

### 3.4. Surprisal Estimation

We investigate effects of three measures of surprisal: (i) *unigram surprisal*, (ii) *context-dependent surprisal* that takes into account preceding linguistic material within the utterance and (iii) *surprisal reduction*, which quantifies how much inserting a word reduces the surprisal of the following word. In our data set, unigram surprisal models the likelihood of a word to appear given a particular extralinguistic context, since we estimated it individually for each script. Our measure of context-dependent surprisal is similar to the approach to surprisal by Hale (2001), but it is robust with respect to the circularity issue that results from estimating surprisal on elliptical data. We use these first two measures to investigate whether, as our account predicts, predictable words are more often omitted. Our third predictor, surprisal reduction, allows us to investigate the second prediction of UID, i.e., whether words are more likely to be realized when they reduce the surprisal of following material. Previous research on function words (e.g., Levy and Jaeger, 2007; Jaeger, 2010; Lemke et al., 2017) addressed this question by estimating the surprisal of the word following the target word on a modified corpus, from which all instances of the target word, e.g., relative pronouns or articles, had been omitted. This approach is not applicable to fragments though, because in principle all parts of speech can be omitted in fragments. Therefore, we developed a measure of surprisal reduction that quantifies to what extent inserting or omitting a target word *w_*i*_* before its successor *w_*i*+1_* reduces the surprisal of *w_*i*+1_* in a particular context.

#### 3.4.1. Unigram Surprisal

We estimate the *unigram surprisal* of each word in the preprocessed data with unigram language models with Good-Turing discount on the preprocessed data that we trained using the SRILM toolkit (Stolcke, 2002). We trained an individual language model on the data for each script separately, because this allows us to interpret surprisal as conditioned on the script-based situation, i.e., on the extralinguistic context (4). For instance, it will show how likely a word like pasta is at a particular position in an utterance produced given the pasta script, without taking potentially preceding words into account.


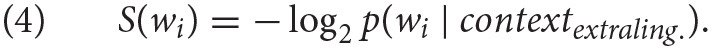


#### 3.4.2. Context-Dependent Surprisal

We use a novel method to estimate *context-dependent surprisal*, which takes into account preceding words in addition to extralinguistic context. In previous research, effects of linguistic context on surprisal were often measured with bigram or higher order *n*-gram models, which return a word's likelihood given the previous *n* − 1 words. Currently there are more advanced language modeling techniques that take into account larger parts of linguistic context (Iyer and Ostendorf, 1996; Oualil et al., 2016a,b; Singh et al., 2016; Grave et al., 2017; Khandelwal et al., 2018; Devlin et al., 2019). However, training even those advanced models on corpus data brings along a circularity issue observed by Levy and Jaeger (2007, p. 852): If predictable words are omitted more often than unpredictable ones, their corpus frequency is not proportional to their predictability. This problem could be addressed by training the model on the enriched data set, i.e., after ellipsis resolution, but this option results in further issues. For instance, consider the case of the fragment (5-a), which is derived from the sentence (5-b) by omitting the NP pasta. An *n*-gram model trained on the complete sentence would estimate the surprisal of pot.goal as *p*(*pot.*goal | *pour pasta*), but this is psychologically implausible: Since the word pasta is not included in the actual linguistic context, it cannot affect the likelihood of pot.goal.


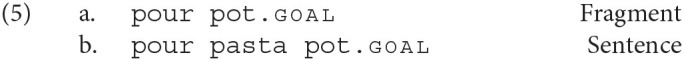


Therefore, we estimate context-dependent surprisal (and surprisal reduction, see below) with a method based on the approach by Hale (2001), who derives surprisal from the work done by a fully parallel parser. The parser rejects all parses that are compatible with the input before but not after processing a word, and the processing effort for that word is proportional to the probability mass of the discarded parses: The larger the total probability mass of the rejected parses is, the higher is the surprisal of this word. Hale (2001) calculates the surprisal of a word *w_i_* as the log ratio between the prefix probability α, i.e., the total probability mass of the parses compatible with an input, before and after processing *w_i_*, as shown in equation 1.

(1) $$\begin{array}{l} \text{S}\left(w_{i}\right)=\log_{2}\frac{\alpha_{\text{i}-1}}{\alpha_{\text{i}}} \end{array}$$

The application of this approach to a data set requires to know the set of possible parses, i.e., the possible structures in a language, and their respective likelihood. Hale (2001) uses a probabilistic context-free grammar (PCFG) to obtain both the set of possible parses and to calculate their probabilities. He does not discuss fragments, but in principle fragments like pour pot.goal could be accounted for by the rule in (6-a), whose likelihood can be estimated from a corpus. However, this would raise a circularity issue which is similar to the one discussed above. If speakers often omit objects like *the pasta* in such fragments, the rule corresponding to the complete structure (6-b) will have a lower probability than (6-a) and the NP consequently be assigned a high surprisal in this context, rather than the low one that motivates its frequent omission.


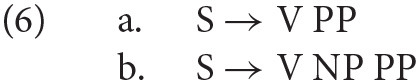


The first main difference between Hale's approach and ours is that rather than using a PCFG to estimate the likelihood of structures, we assume that the set of possible complete structures is equal to the set of complete structures in our enriched production data set, i.e., the pre-processed data set after the reconstruction of omissions. Since this set is finite, it is straightforward to determine each complete structure's probability. The second main difference to Hale's method concerns the question of which complete structures are excluded by an input. Hale (2001) rules out all parses that are not identical to the input up to the currently processed word. For instance, a fragment like (7-a) is compatible with the complete structure in (7-b), but not with (7-c), because it does not start with the word *pour*.


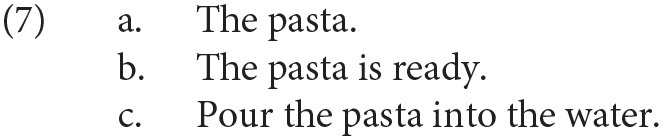


However, the fragment in (7-a) can be derived from both (7-b,c) by omission. Therefore, we do not require the input and the parse to be identical to be included in the set of parses that are compatible with the input, but for the input to be potentially derived by omissions from the parse. More technically, we allow for an arbitrary number of omissions to occur before, between and after all words in the our enriched representations when checking whether the current input is compatible with a particular parse.

In what follows, we illustrate how our approach allows us to estimate the surprisal of omitted and realized words at the case of the fragment pour pot.goal in (5-a), for which we assume the underlying complete structure in (5-b). For expository purposes, we assume the hypothetical probability distribution over complete structures in (8), but the approach works identically for the actual production data.


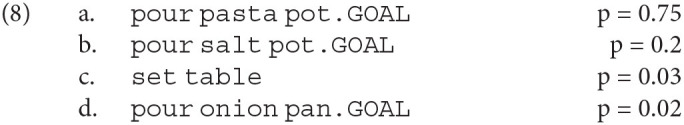


Given this probability distribution, the surprisal of pour at the onset of the utterance can be estimated just like Hale (2001) proposes. Before any input is processed, no parse is excluded, hence the prefix probability α_*onset*_ = 1. Processing the word pour rules out (8-c), because it is the only complete structure that does not contain the word pour, so that α_*pour*_ = 0.97. The surprisal of pour at the utterance onset is then calculated as shown in equation 2.

(2) $$\begin{array}{l} \text{S}\left(pour|onset\right)=\log_{2}\frac{\alpha_{\text{onset}}}{\alpha_{\text{pour}}}=\log_{2}\frac{1}{0.97}=0.04\;bits \end{array}$$

Similarly, the surprisal of the omitted word pasta given pour is equivalent to the ratio of the cumulated probability mass of all parses that contain the word pour, i.e., (7-a,b,d) and those which contain the word pour followed by pasta, i.e., (8-a). Since α_*i*−1_ = 0.97 and α_*i*_ = 0.75, the context-dependent surprisal of pasta is calculated as shown in equation 3. Note that this surprisal estimate is not affected by the actual omission of the word pasta, because the prefix probabilities are calculated based on the complete structures in (8) alone. Therefore, it is not affected by the circularity issue discussed above and can be used as a predictor of omission in our statistical analysis.

(3) $$\begin{array}{l} \text{S}\left(pasta|pour\right)=\log_{2}\frac{\alpha_{\text{pour}}}{\alpha_{\text{pasta}}}=\log_{2}\frac{0.97}{0.75}=0.37\;bits \end{array}$$

In order to calculate the surprisal of pot.goal in (5-a), we compare the probability mass of all parses that contain pour, i.e., (8-a,b,d) and the probability mass of the parses that contain pot.goal somewhere after pour (8-a,d). Since pasta has been omitted, the current input pour pot.goal can be derived from both of the complete structures in (8-a,d) by omission. Again, the surprisal of pot.goal is calculated by applying Hale's formula, as shown in equation (9). In this case the surprisal estimate is affected by the omission of the word pasta that could precede the target word pot.goal. This is desirable, because it would not be psychologically realistic to assume that a hearer who processes the reduced utterance relies on words that have been omitted to estimate the surprisal of following ones.


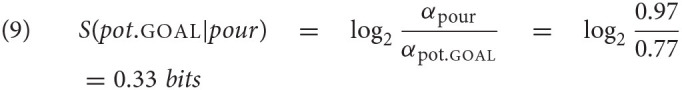


Taken together, our approach avoids the circularity issue caused by omissions of frequent words in the data used for surprisal estimation because it relies on nonelliptical representations for calculating the prefix probabilities. It is also psychologically realistic because it quantifies the work done by the parser incrementally and omitted words in the context of a target word cannot affect the target word's probability.^10^

#### 3.4.3. Surprisal Reduction

Our last measure is *surprisal reduction*, which quantifies how much inserting *w_i_* reduces the surprisal of *w_i+1_*. Whereas, context-dependent surprisal quantifies the processing effort of a *w_*i*_* itself and thus allows us to investigate whether predictable words are more often omitted, surprisal reduction can show us whether the degree to which inserting a word *w_*i*_* reduces the surprisal of the following word *w_i+1_* also constrains the likelihood of the omission of *w_*i*_*. Some of the previous studies investigating UID effects on reduction (e.g Levy and Jaeger, 2007; Frank and Jaeger, 2008) used the *n*-gram surprisal of *w_*i*+1_* to investigate this prediction, but in case of our study this was not reasonable: UID does not predict arbitrary insertions before unpredictable words, but that insertions are only useful when they reduce the surprisal of unpredictable words.^11^ For this purpose, we calculate the ratio between the prefix probability at *w_i+1_* if *w_i_* has been realized and the prefix probability at *w_i+1_* if *w_i_* has been omitted (10).


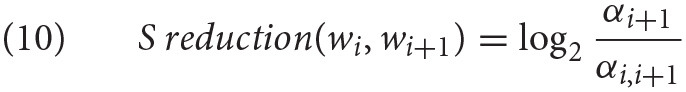


Again, we illustrate this idea at the simplified pasta script by quantifying how much inserting pasta before pot.goal in a fragment pour pot.goal reduces the surprisal of pot.goal as compared to the omission of pasta. In this case, the probability mass of all parses that contain the words put and pot.goal in this order, with potentially intervening material (i.e., 7a,b), is compared to the ratio of those parses that additionally contain pasta between these words (8-a). Since α_*i*_ = 0.95 and α_*i,i*+1_ = 0.75, inserting pasta reduces the surprisal of pot.goal by log_2_(0.95/0.75) = 0.34 bits.

### 3.5. Results

#### 3.5.1. Data Set Statistics

**Figure 3:**
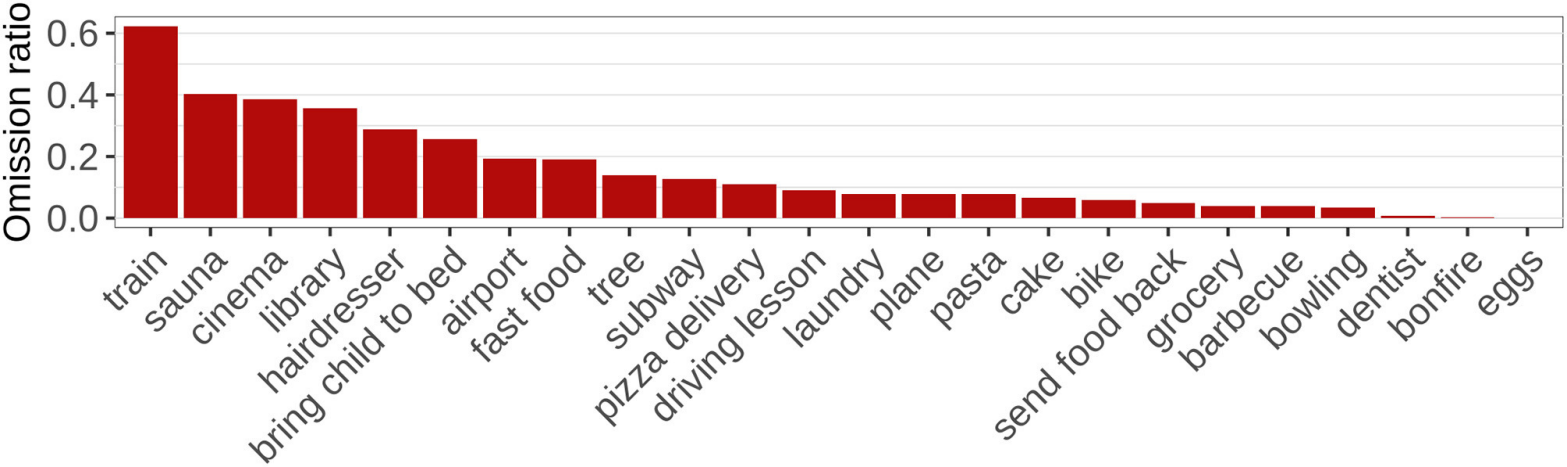
Ratio of omission across scripts.

The preprocessed data set comprises a total of 2.409 sentences consisting of 6.816 primitive expressions (“words”). 1.052 (15.43%) of these words had been omitted in the original data set. As Figure 3 shows, scripts differ to a large extent with respect to the ratio of words that were omitted. For instance, in the train script 62.3% of the words were omitted, whereas there are no omissions at all in the cooking scrambled eggs script.

The low ratio of omission in some scripts raises the question of whether this variation occurs due to properties of the situation which might override the predictions of our information-theoretic account, or whether our account predicts such variation. For instance, sentences might be perceived as more polite than fragments, so that in situations where politeness matters there might be a preference for full sentences which is the result of information-theoretic considerations. In contrast, the responses collect for a script might differ between scripts in their degree of variation. If there are only few different words in the data for a scenario, and/or the probability distribution over these words is skewed, i.e., some words are much more likely than others, an average word in that data will be more predictable. Since we expect that a word's probability predicts the likelihood of its omission, a varying omission rate between scenarios could result from different probability distributions over words.

**Figure 4:**
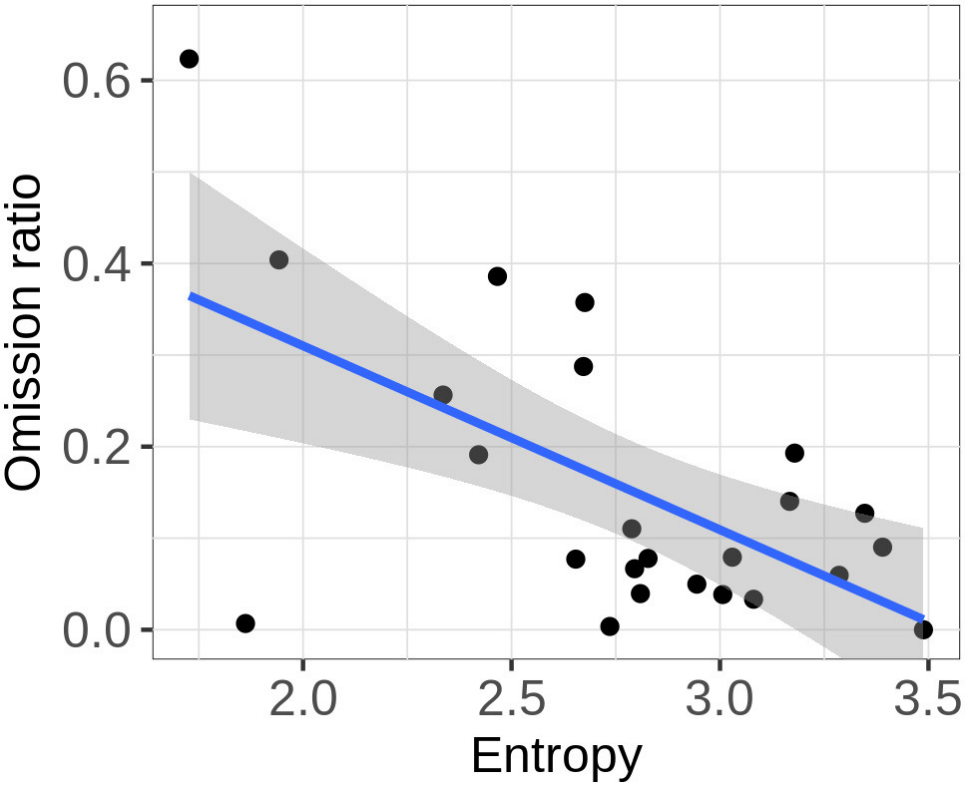
Ratio of omission across scripts as a function of the entropy in the probability distribution over words in the data for that script. Each data point represents one script.

We test this hypothesis by investigating whether the ratio of omission is higher in scripts with a higher degree of variation between words. For this purpose, we estimate the entropy in the probability distribution over words in the enriched data set for each script after preprocessing and ellipsis resolution. Following (Shannon, 1948), the entropy, which quantifies the degree of uncertainty about the outcome of a random variable, is defined as shown in equation 4. It equals 0 when there is no variation in the data, i.e., when there is only one possible word, which has a probability of 1, and it is maximal when all words in the data are equally likely. Furthermore, it increases as the number of different words in the data grows. Figure 4 suggests that the entropy in the data for a script is indeed related to the rate of omissions: The omission rate seems to be higher in scripts with a low entropy. This is confirmed by a linear regression (R Core Team, 2019) which shows that entropy has a significant effect on the ratio of omission (*F*_(1)_ = 12.49, *p* < 0.01).^12^

(4) $$\begin{array}{l} H\;=\;-K\;\overset{n}{\underset{i=1}{\sum }}\;p_{i}\;log\;p_{i} \end{array}$$

#### 3.5.2. Statistical Methodology

We analyzed the data with mixed effects logistic regressions (lme4, Bates et al., 2015) in R (R Core Team, 2019). Our regressions predict the actual omission of the words in the enriched data set from the three surprisal measures that we introduced in section 3.4. We investigate the effects of these predictors individually with three separate analyses. Even though it would have been desirable to test for effects of these predictions in a single analysis in order to tease apart effects of linguistic and extralinguistic context on predictability, Table 1 shows that the measures are correlated with each other, and context-dependent surprisal is particularly strongly correlated with the other two measures.

**Table 1:** Correlations between surprisal predictors.

**Predictors**	***r*^2^**	***t*-value**	***p*-value**
Unigram, context	0.65	70.06	<0.001
Unigram, reduction	0.48	37.99	<0.001
Context, reduction	0.62	54.0	<0.001

Therefore, we first conduct two analyses that test for effects of unigram and context-dependent surprisal on the complete data set. In a third analysis we take into account unigram surprisal and surprisal reduction. This last analysis investigates only non-final words, since the last word in an utterance lacks a successor, whose predictability its insertion or omission could affect. In all analyses we conducted model comparisons with likelihood ratio tests computed with the anova function in R and maintained only those effects in the model that significantly improved model fit.

#### 3.5.3. Avoid Troughs: Unigram Surprisal and Context-Dependent Surprisal

**Figure 5:**
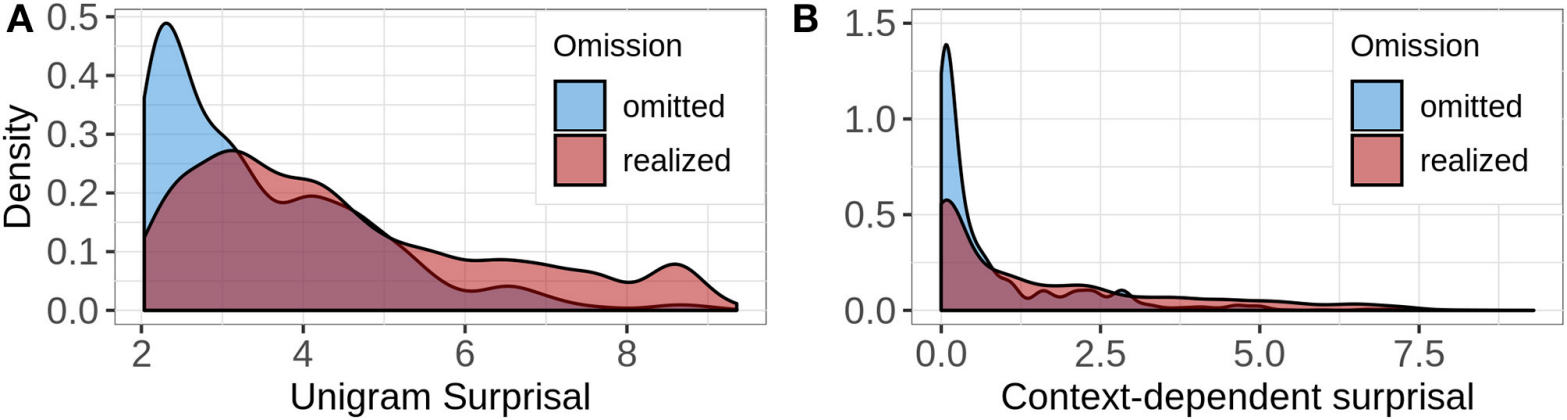
The density plots illustrate the distribution of words that were originally omitted and those originally realized across the surprisal ranges. The left facet **(A)** shows the distribution for unigram surprisal and the right facet **(B)** shows the distribution for context-dependent surprisal.

Figure 5 shows how the omitted and the realized words are distributed across the range of unigram surprisal (left facet) and context-dependent surprisal (right facet). For both predictors, the originally omitted words appear toward the lower end of the scale, whereas the realized words seem to have a higher surprisal on average. The distribution for context-dependent surprisal is highly skewed, because in our highly standardized data set sometimes a single word fully disambiguates between two utterances and consequently, all words that appear later in the utterance have a surprisal of 0. For instance, in case of our simplified example in (8) above, put salt pot.goal is the only utterance that contains the word salt, so that pot.goal has a probability of 1 and a surprisal of 0 in this context.

The models in the analyses of unigram surprisal^13^ and context-dependent surprisal^14^ contained by-script random intercepts and slopes for surprisal and by-subject random intercepts. Tables 2, 3 summarize the final models for the analyses. Both of the analyses reveal significant main effects of the respective predictor, which support our hypothesis that predictable words are more likely to be omitted. Unlike we expected though, the effect for unigram surprisal (χ^2^ = 7.39, *p* < 0.01) is stronger than that of context-dependent surprisal (χ^2^ = 4.86, *p* < 0.05). In principle we would expect the opposite pattern, since previous research on information-theoretic constraints on omissions has found robust predictability effects driven by linguistic context. In the case of our data, the relatively large number of words that are assigned a context-dependent surprisal of 0 and that were nevertheless realized might account for this pattern. Even though these words are fully predictable in our enriched data set, they are not necessarily equally predictable to an actual speaker, for instance because of different lexicalizations which we merged during preprocessing. Furthermore, our data set contains only those utterances that appeared to be the most likely ones by at least one of our participants, but not utterances that everybody considers to be relatively unlikely. An actual speaker however must reserve some probability mass to those utterances as well, so she would not assign as many words a surprisal of 0 as our account does, and consequently choose not to omit some of these words. Therefore, we expect to find stronger effects of context-dependent surprisal in case of larger and more diverse data sets.

**Table 2:** Fixed effects in the final GLMM investigating the effect of UNIGRAMSURPRISAL on OMISSION.

**Predictor**	**Estimate**	**SE**	**χ^2^**	***p*-value**	
UNIGRAMSURPRISAL	–0.337	0.117	7.39	<0.01	

**Table 3:** Fixed effects in the final GLMM investigating the effect of CONTEXTSURPRISAL on OMISSION.

**Predictor**	**Estimate**	**SE**	**χ^2^**	***p*-value**	
CONTEXTSURPRISAL	–0.28	0.126	4.86	<0.05	

#### 3.5.4. Avoid Peaks: Surprisal Reduction

The analysis that includes surprisal reduction and unigram surprisal was conducted on a subset of the data that contained those non-final words that were not followed by an ellipsis (55.51% of the total data). Only these words have a successor which is not omitted and whose surprisal might be constrained by the omission or realization of the preceding word. The full model contained main effects of both predictors as well as their interaction, as well as random intercepts for subjects and scripts.^15^ The final model contains only the main effects, both of which support our predictions (see Table 4). The effect of unigram surprisal (χ^2^ = 10.39, *p* < 0.01) replicates the effect found in the analysis of the full data set. The effect of surprisal reduction (χ^2^ = 27.03, *p* < 0.001) shows that, as we expected, words that reduce the surprisal of the following word more strongly are more likely to be realized. There is no significant interaction between both predictors (χ^2^ = 0.01, *p* > 0.9).

**Table 4:** Fixed effects in the final GLMM investigating effects of both UNIGRAMSURPRISAL and SURPRISALREDUCTION.

**Predictor**	**Estimate**	**SE**	**χ^2^**	***p*-value**
UNIGRAMSURPRISAL	–0.151	0.046	10.39	<0.01
SURPRISALREDUCTION	–0.349	0.07	27.03	<0.001

## 4. Discussion

In this article we propose an information-theoretic account as an answer to the previously underexplored question of why speakers use fragments, when they prefer a fragment over the corresponding complete sentence, and if they do so, which fragment is ultimately selected. The empirical predictions of our account are supported by the results of a production task: First, speakers tend toward omitting words that are predictable in context in order to make a more efficient use of the hearer's cognitive resources. Second, speakers tend toward inserting words that could be omitted but that increase the predictability of the following word. This reduces peaks in the information density profile of the utterance, which would be otherwise likely to exceed the resources available to the hearer.

Our study provides the first systematic investigation of why speakers use fragments. Previous research on fragments investigated their syntactic properties and licensing conditions, and pursued almost exclusively theoretical approaches. As we observed in the introduction, information structure-based syntactic accounts of fragments (Merchant, 2004; Reich, 2007; Weir, 2014; Ott and Struckmeier, 2016; Griffiths, 2019) explain under which circumstances fragments can be used, but not why speakers choose to produce a fragment or a complete sentence. Our information-theoretic account provides a potential solution to this issue: Speakers prefer to use fragments when the omission of words that are obligatory in full sentences (like finite verbs and their arguments), which results in fragments, optimizes the form of the utterance with respect to the processing resources which are available to the hearer.

Our results are partially in line with other theories of probability-driven reduction of linguistic expressions, like the availability-based production theory (Ferreira and Dell, 2000) or a source coding account (Zipf, 1935; Pate and Goldwater, 2015). Even though not all of these theories have been applied to fragments, they make predictions on the distribution of omissions that can result in fragments. However, none of these theories covers the complete empirical picture, that is, the preferences to omit predictable words and to insert redundancy when this increases the likelihood of following unpredictable words.

Availability-based production (e.g., Bock, 1987; Ferreira and Dell, 2000) explains some predictability effects with speaker-centered production difficulties: Retrieving words from memory is effortful, and the retrieval of unpredictable words requires more effort, i.e., time. Since speakers intend to avoid disfluencies which result from the effortful retrieval of unpredictable words, they insert optional words before unpredictable material in order to keep speech fluent. Therefore, availability-based production predicts the insertion of optional words before unpredictable words, but not that predictable words are more likely to be omitted.

The opposite holds for a source coding account, which takes into account only properties of the *source* in the model of communication assumed by Shannon (1948), i.e., the frequency of expressions. A system that assigns a unique utterance to each message is more efficient if it assigns longer utterances to rare meanings and reserves the shortest encodings for the most frequent meanings. This predicts more likely utterances to be more often reduced, as Lemke et al. (2021) and we show, but not that speakers *insert* redundancy to *reduce* processing effort.

The main difference between the predictions of the game-theoretic account in Bergen and Goodman (2015) and our UID-based account is that according to game-theoretic accounts there is no upper bound to the densification of utterances, like channel capacity. In practice, if game-theoretic models are applied to larger and more diverse data sets, they might indirectly predict a similar effect though: Fragments like (11-a) can communicate different meanings, and if a particular meaning is more likely in a situation, like (11-b) as compared to (11-c) in our taxi scenario, the game-theoretic approach also predicts that speakers use the fragment to refer to the predictable utterance. Due to the high prior probability of (11-b) given (11-a), the hearer will choose this interpretation of the fragment and the speaker will in turn rather produce a complete sentence if she wishes to communicate the more unlikely message in (11-c). Empirically comparing game-theoretic models of fragment usage to our UID-based account would require a more precise formulation of how to derive the set of alternative utterances and messages to be considered in a situation and how the cost of producing an utterance is derived.


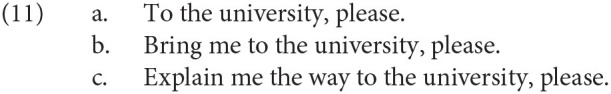


Our UID-based account predicts both the omission of predictable words and the insertion of additional redundancy before unpredictable words that the analysis of our production data revealed. Other theories that have been proposed in the literature to account for other optional omission phenomena, like availability-based production and source coding, or specifically applied to fragments, like the noisy channel model by Bergen and Goodman (2015), can only explain part of the data, but not the complete empirical pattern.

From the broader perspective of information-theoretic research on the choice between linguistic encodings, our study extends previous evidence for predictability effects on optional omissions in two ways. First, we present evidence for such effects on content words like nouns and verbs, whereas previous work focused on semantically relatively empty closed-class function words. This requires a modified approach to surprisal estimation, since *n*-gram models trained on regular corpus data suffer from a circularity issue and removing all target words from the corpus [following Levy and Jaeger (2007)] is not possible for content words. Second, we find predictability effects based on extralinguistic context on omissions. Most of the previous studies only took local linguistic context into account. Instead, we provide evidence for effects of script knowledge on predictability and consequently omissions.

Even though our study was relatively resource-intensive due to the large amount of manual preprocessing, there are several ways in which it could be extended in future research. First, we observed that effects of linguistic context are not as strong as we expected in our data set, and this might be in part due to the high degree of standardization and the amount of utterances per script, which is relatively small as compared to corpora like those used in previous studies on UID effects on omissions. Future research that relying more strongly on automatized procedures might be able to process larger data sets in a similar way and yield more fine-grained results. Second, our data set is probably a close approximation to hearers' expectations about what will be said in the situations described in our context stories. However, this expectation might differ to some extent from expectations developed by hearers in such situations, because we asked only for a single most likely utterance provided by each participant. There might be overall less likely, and yet salient, utterances, which hearers assign a probability, but which is not reflected in our data. A possible solution for this issue would consist in asking participants to provide a series of utterances and to specify the relative likelihood of each one, be it in terms of absolute probability or by ranks. Third, since we are interested in the usage of fragments, we deliberately preprocessed our data so that it contained only words whose omission would result in a fragment or that are obligatory in standard sentences, like main verbs and their arguments. As we noted above, in principle UID also predicts that likely adjuncts, e.g., temporal or local adverbials, will be omitted when they are predictable. This issue could be empirically investigated with a method that is similar to ours, but it would require an even more extensive preprocessing approach in order to neatly reconstruct all implicit adverbials.

Taken together, our research makes three main contributions: First, we propose an information-theoretic account as an answer to the question of why and when fragments are used. The two central predictions of our account are supported by our production study: Predictable words are more often omitted and additional redundancy is inserted in order to reduce the processing effort of following words. Second, we extend previous evidence for information-theoretic processing constraints on linguistic encoding choices, and third, our methodological approach might be also applied to other omission phenomena.

## Data Availability Statement

The original contributions generated for the study are included in the article/Supplementary Material, further inquiries can be directed to the corresponding author/s.

## Ethics Statement

The studies involving human participants were reviewed and approved by Ethics Committee of the Deutsche Gesellschaft für Sprachwissenschaft (German Society for Language Science). Written informed consent for participation was not required for this study in accordance with the national legislation and the institutional requirements.

## Author Contributions

RL conducted the experiment, supervised the annotation of the production data, developed the surprisal estimation methods, conducted the statistical analyses, and wrote the initial version of the article. IR acquired the project funding and conceptualized the overarching research program and goals. LS created software for pre-processing the corpus on which the experimental materials are based. HD and IR managed and supervised the research activities. IR, LS, and HD critically revised previous versions of the article. All authors contributed to the article and approved the submitted version.

## Conflict of Interest

The authors declare that the research was conducted in the absence of any commercial or financial relationships that could be construed as a potential conflict of interest.

## Footnotes

^1^In the theoretical literature there is no agreement on a definition of the notions *nonsentential utterance* or *fragment*. Researchers diverge in particular with respect to the question of which elliptical utterances are categorized as fragments and with respect to the presence or absence of an explicit antecedent. As for the first question, in this article we restrict ourselves to DP fragments, which are analyzed as fragments by all accounts of fragments. As for the second question, some researchers (see e.g., Merchant, 2004; Barton and Progovac, 2005) do not distinguish between fragments that occur discourse-initially and those that have a linguistic antecedent, like short answers (but cf. Klein, 1993; Reich, 2011). We avoid this debate by investigating only uncontroversial discourse-initial fragments.

^2^But cf. Kravtchenko (2014); Schäfer (2021).

^3^In the literature there is no consensus on which expressions are classified as fragments. For instance, Barton and Progovac (2005) discuss the omission of articles and prepositions in an article on the syntax of fragments, whereas e.g., Merchant (2004) takes into account mostly bare DPs and PPs. In this article we avoid this issue by testing only discourse-initial bare DP fragments, which are classified as fragments by all theories thereof.

^4^This view differs from the discussion of channel capacity in related work (Levy and Jaeger, 2007; Jaeger, 2010), which emphasizes on the role of noise in determining channel capacity. Shannon (1948) shows that if the channel is noisy, the transmission rate can be increased up to channel capacity without increasing the noise ratio. Attempts to increase the transmission rate beyond channel capacity however reduces the actual transmissions rate because of an increased noise rate. The noise-based and the processing effort-based interpretations of channel capacity do not contradict each other, and with respect to our study, their predictions are identical. However, we tentatively assume that on the word level our processing account is more appropriate. Falsely perceiving a phoneme like /p/ as /b/ due to noise is relatively likely, whereas perceiving a word like *Mary* as *John* under normal communication conditions is much more unlikely, since they differ in a larger set of phonemes.

^5^Whether inserting words can only increase or also reduce the likelihood depends on the method used for surprisal estimation. Following the approach proposed by Hale (2001) words can only reduce the surprisal of following material, but the entropy-based method by Levy (2008) predicts that the insertion of preceding material can also increase the surprisal of a target word.

^6^The stories were originally used in the rating study in Lemke et al. (2021), we refer the reader there for more details on this procedure.

^7^For instance masculine singular DPs distinguish four cases in German, whereas feminine singular and plural DPs have a partially syncretic paradgim (i).

^8^An anonymous reviewer pointed out that much of the previous research has focused on word probabilities, so that the surprisal difference between frequent and infrequent synonyms would affect the predictions of the theory. This is in principle correct, but we decided to pool synonyms because otherwise the unambiguous resolution of omissions would be impossible.

^9^In principle, it would be interesting to look into the implicit or explicit realization of adjuncts from a UID perspective. We would expect that highly predictable adverbials are more often omitted, too.

^10^Note that our approach is not technically identical to the definition of surprisal in the literature, because the surprisal values assigned to the words that follow *w_*i*−1_* do not necessarily sum up to 1. The reason for this is that individual parses can contribute to the probability mass of not only a single word *w_*i*_*, but also to that of a word *w_*j*_*, which appears after *w_*i*_* in the parse, provided that *w_*i*_* has been omitted. Even though our approach loses this mathematical property of the original definition of surprisal in the literature, the probability estimate that we propose is in line with the insight by Hale (2001) that processing effort is proportional to the probability mass of the parses that are compatible with an input.

^11^This concern does not apply to the studies cited here. Levy and Jaeger (2007) looked into effects of additional processing effort due to syntactic surprisal and Frank and Jaeger (2008) investigated contractions, so their study is not affected by the issues concerning omissions.

^12^We also tested for a potential effect of raw lexicon size, which also predicts the rate of omission (*F*_(1)_ = 6.18, *p* < 0.05). However, if entropy is included in the model, the effect of lexicon size is no longer significant (*F*_(1)_ = 6.18, *p* < 0.05).

^13^Ellipsis ~ UnigramS + (1+UnigramS | Script) + (1 | Subject).

^14^Ellipsis ~ ContextS + (1+ContextS | Script) + (1 | Subject).

^15^Ellipsis ~ UnigramS * SReduction (1 | Script) + (1 | Subject).
